# Performance Evaluation of a Prototype Architect Antibody Assay for Babesia microti

**DOI:** 10.1128/JCM.00460-18

**Published:** 2018-07-26

**Authors:** Kevin Cheng, Kelly E. Coller, Christopher C. Marohnic, Zachary A. Pfeiffer, James R. Fino, Randee R. Elsing, Janet Bergsma, Marilee A. Marcinkus, Alak K. Kar, Orlando H. Gumbs, Kathy S. Otis, Jeffrey Fishpaugh, Phillip W. Schultz, Mark R. Pope, Alfredo R. Narvaez, Susan J. Wong, Susan Madison-Antenucci, Thomas P. Leary, George J. Dawson

**Affiliations:** a Abbott Laboratories, Abbott Park, Illinois, USA; b The Wadsworth Center, New York State Department of Health, Albany, New York, USA; c The Department of Biomedical Sciences, The School of Public Health, The University at Albany, Albany, New York, USA; University of Texas Medical Branch

**Keywords:** *Babesia microti*, immunoassay

## Abstract

The tick-borne protozoan Babesia microti is responsible for more than 200 cases of transfusion-transmitted babesiosis (TTB) infection in the United States that have occurred over the last 30 years. Measures to mitigate the risk of TTB include nucleic acid testing (NAT) and B. microti antibody testing. A fully automated prototype B. microti antibody test was developed on the Architect instrument. The specificity was determined to be 99.98% in volunteer blood donors (*n* = 28,740) from areas considered to have low endemicity for B. microti. The sensitivity of the prototype test was studied in experimentally infected macaques; a total of 128 samples were detected as positive whereas 125 were detected as positive with an indirect fluorescent antibody (IFA) test; additionally, 83 (89.2%) of the PCR-positive samples were detected in contrast to 81 (87.1%) using an IFA test. All PCR-positive samples that tested negative in the prototype antibody test were preseroconversion period samples. Following seroconversion, periods of intermittent parasitemia occurred; 17 PCR-negative samples drawn in between PCR-positive bleed dates tested positive both by the prototype test (robust reactivity) and IFA test (marginal reactivity) prior to the administration of therapeutic drugs, indicating that the PCR test failed to detect samples from persistently infected macaques. The prototype assay detected 56 of 58 (96.6%) human subjects diagnosed with clinical babesiosis by both PCR and IFA testing. Overall, the prototype anti-Babesia assay provides a highly sensitive and specific test for the diagnosis of B. microti infection. While PCR is preferred for detection of window-period parasitemia, antibody tests detect infected subjects during periods of low-level parasitemia.

## Abstract (precis)

The tick-borne protozoan Babesia microti is responsible for more than 200 cases of transfusion-transmitted babesiosis (TTB) infection in the United States that have occurred over the last 30 years. Measures to mitigate the risk of TTB include nucleic acid testing (NAT) and B. microti antibody testing.

## INTRODUCTION

Babesia microti, an intraerthrocytic protozoan parasite, is a member of the Babesia genus (phylum Apicomplexa, order Piroplasmida) primarily transmitted to humans through the bite of the deer tick (Ixodes scapularis), endemic to the northeastern and upper-midwestern regions of the United States. Though most B. microti infections are asymptomatic, in some cases, mild to severe malaria-like illness (babesiosis) characterized by fever, chills, myalgia, fatigue, hepatosplenomegaly, and hemolytic anemia have been reported (1). The symptoms can be severe, especially among splenectomized, immunocompromised, or elderly individuals, with mortality rates up to 5% (2, 3). Since January 2011, when babesiosis became a nationally notifiable disease, the CDC has been monitoring the number of cases. Between 2011 and 2014, the number of babesiosis cases reported ranged from 911 to 1,761 cases annually, with 2013 and 2014 representing the largest numbers of cases at 1,761 and 1,744, respectively (4). For 2014, 94% of the babesiosis cases were reported from seven states (New York, Connecticut, Massachusetts, Rhode Island, New Jersey, Minnesota, and Wisconsin) considered to be areas of endemicity for B. microti (4).

In the early 1980s, it was recognized that blood donors harboring B. microti can transmit the parasite to recipients (5). A subsequent study reported 159 cases of transfusion-transmitted babesiosis (TTB) due to B. microti and 3 cases due to Babesia duncani between 1979 and 2009 (6). Approximately 87% of the TTB index cases occurred in the seven states where B. microti is endemic. A more recent compilation of TTB cases indicates that there have been more than 256 cases reported (7). The estimated risk of TTB in selected counties of endemicity is 1 per 101,000 donations, with greater risk in counties of high endemicity (8). The number of transfusion-associated B. microti cases is likely much higher as many cases are either not recognized or not reported. Currently, B. microti is the highest-ranking transfusion-transmitted pathogen for which there is no blood donor screening test in the United States, and it is the leading cause of transfusion-associated death attributed to an infectious pathogen (9). Additionally, organ transplantation has been implicated in B. microti transmission as recipients of renal allografts from an untested organ donor have transmitted B. microti (10).

Currently, there are no licensed molecular or serologic tests to screen blood donors for B. microti. The most widely used method for diagnosis of infection is an indirect fluorescent antibody (IFA) test which employs B. microti parasitized erythrocytes as the antigen source (11–13). While the IFA test is useful, the assay is labor-intensive, not standardized or automated, and not easily adaptable to modern blood screening practices. The IFA assay has been estimated to have 88 to 96% sensitivity and 90 to 100% specificity (11), which may not meet current expectations for blood screening (14). Tests for the detection of active babesiosis include nucleic acid tests (NATs) and blood smear tests. Blood smear tests are not as sensitive as molecular tests and are not suitable for blood screening. Molecular tests target the 18S rRNA gene of B. microti in infected whole red blood cells (15–19). It is estimated that less than 1% of erythrocytes are parasitized early in the course of infection, and the proportion can vary throughout infection (20), with more cases detected via molecular testing than by blood smear.

Two investigational assays (the Immunetics enzyme immunoassay [EIA] and Imugen arrayed fluorescence immunoassay [AFIA]), designed to detect antibodies to B. microti, are under consideration for use in blood screening. Seroprevalence studies using these investigational assays in areas of endemicity and nonendemicity showed rates ranging from 0.28 to 0.75% and from 0.025 to 0.13%, respectively, with corresponding specificities ranging from 99.93 to 99.98% (21, 22). From recent studies, no cases of TTB were observed in regions that implemented serologic testing of blood donors or both molecular and serologic testing while in other areas of endemicity where testing was not implemented, TTB was observed (8, 22, 23). These studies suggest that antibody testing may be useful to identify potential parasitemic blood donors and thereby reduce TTB. Molecular testing of whole-blood donations from seropositive samples showed active infection in up to 20% of seropositive samples (8, 21, 22, 24). In one study, AFIA was performed on 89,153 blood donors, resulting in 335 samples that were antibody positive, with 67 being PCR positive; 9 samples were found to be antibody negative but PCR positive (8). Additionally, two seropositive and PCR-negative samples were shown to transmit B. microti upon hamster infection (8). Thus, stand-alone molecular or antibody testing may not be sufficient to ensure a safe blood supply, but this statement will depend on the sensitivity of the molecular test that is being employed. In May 2015, the Blood Product Advisory Committee of the FDA recommended that antibody screening be performed nationwide year round and that molecular testing be performed only in the states of high endemicity (14).

We present a research prototype serology test for the detection of both IgM and IgG antibodies to B. microti on the high-throughput Architect immunoassay platform. Specificity testing was performed on 28,740 plasma and serum donors from areas of nonendemicity and was found to be 99.98%. The sensitivity of the prototype was compared to that of the IFA test. The detection between the two assays correlated on well-characterized samples and serial bleeds from a macaque model of TTB. Automated platforms, such as those described in the study, may be useful for performing expanded studies to determine seroprevalence and for potentially screening blood donors for antibodies to B. microti.

## MATERIALS AND METHODS

### Samples.

A total of 28,740 volunteer blood donor plasma and serum samples were sourced from the United States (ProMedDx, Norton, MA; Gulf Coast Regional Blood Center, Houston, TX; the American Red Cross, Gaithersburg, MD) for specificity testing. Samples were sourced from areas of nonendemicity for B. microti (southwestern United States, Texas, and Montana). Chronic and active Lyme disease-diagnosed samples were purchased from ProMedDx (Norton, MA, USA).

Dilutional sensitivity was determined using the Center for Biologics Evaluation and Research (CBER) Babesia antibody reference panel (lots 3001 to 3009). Briefly, eight of the nine panel samples were comprised of dilutions of a pool of human plasma samples that were reactive to B. microti antigens in an IFA assay. One panel specimen was a plasma sample that was negative for B. microti antibodies by IFA assay.

For evaluating performance of the prototype B. microti antibody assay, samples were available from a previously described rhesus macaque model of TTB (25). Briefly, a total of 6 monkeys were experimentally infected with either hamster-passaged (2 macaques, phase 1) or monkey-passaged B. microti (3 macaques, phase 2, and 1 macaque, phase 3) to simulate TTB. The kinetics of parasitemia was monitored by blood smear, quantitative PCR (qPCR), and enhanced (nested) PCR, and the immune response was measured using an IFA assay (25). Serum was collected over 210 days for each macaque, and all animals were treated with atovaquone and azithromycin for 10 days starting on day 122 postinfection. Phase 1 and 3 animals received additional treatment on days 193 and 200, respectively.

Fifty-eight clinical babesiosis samples diagnosed with B. microti infection were provided from the Wadsworth Center (New York State Department of Health [NYSDOH], NY, USA). Samples were diagnosed as B. microti PCR positive and/or IFA assay positive (11, 16). All samples were collected in the New York state area during the 2015 season.

### Prototype B. microti antibody assay.

A direct antibody assay was created for the Architect immunoassay instrument (Abbott Laboratories, Abbott Park, IL) for the detection of antibodies to B. microti (referred to as the prototype assay). The direct assay format is not antibody class specific and detects all antibodies. Briefly, two recombinant proteins derived from the BMN1-9 (26–28) and BMN1-17 (26) open reading frames were coated onto a magnetic microparticle solid phase while two other recombinant proteins comprising the same open reading frames were conjugated with acridinium. During the reaction on the Architect, the sample was incubated with the solid phase and conjugates for 18 min allowing for immunocomplex formation. Following a 4-min washing step, the conjugates in the immunocomplex were triggered to emit relative light units (RLU) and measured using a photon multiplier tube. The resulting number of RLU was divided by a provisional cutoff value determined through specificity testing (below) to result in a signal-to-cutoff (S/CO) value.

### Specificity testing.

A total of 19,532 plasma and 9,208 serum samples were tested in the prototype assay. The mean number of RLU and the standard deviation of the population were determined; a provisional cutoff was set at 5,000 RLU, which is equivalent to 31.6 times the standard deviation of the negative population. The provisional cutoff was applied to the population set, and initially reactive samples (S/CO of ≥1.0) were retested in the prototype assay in duplicate. Repeatedly reactive samples were subjected to supplemental IFA assay testing.

### IFA testing.

Samples identified as repeatedly reactive during specificity testing using the prototype assay were subsequently tested by indirect fluorescent antibody (IFA; Quest Diagnostics, Madison, NJ, USA). An IFA test for clinical babesiosis samples was performed at the Wadsworth Center, New York State Department of Health (NYSDOH) (Albany, NY, USA) (11). IFA testing for the macaque serum was performed as described previously (25). In all IFA assays, samples with titers of ≥1:64 were considered reactive for antibodies to B. microti.

### Molecular testing.

For macaques, quantitative PCR was performed as described previously (25); briefly, a quantitative PCR was developed targeting the 18S rRNA gene, and a standard curve using known parasitemia levels was used to express cycle threshold (*C_T_*) values as the number of parasites per microliter. For clinical babesiosis samples, NYSDOH performed reverse transcription-PCR (RT-PCR) on EDTA-preserved whole-blood samples as previously described (16). Briefly, total DNA was extracted from a 200-μl aliquot of blood and eluted in an equal volume. A real-time PCR assay targeting the 18S rRNA gene of B. microti was performed. The assay has been shown to have a limit of detection of between 10 and 100 gene copies per reaction product, which equates to 5 to 10 parasites per μl of blood (16).

## RESULTS

### Specificity characterization and determination of provisional cutoff.

**FIG 1:**
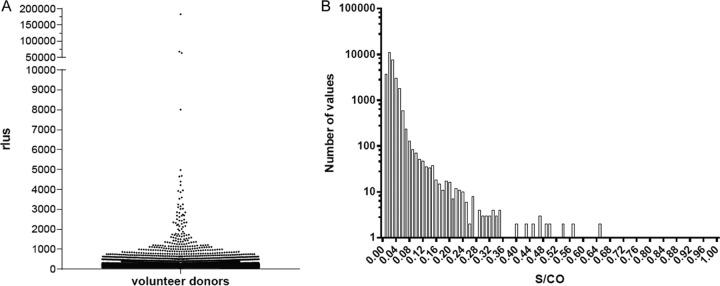
Specificity testing: distribution of volunteer donors. (A) Plotted are RLU values of volunteer donors (*n* = 28,740) from areas of nonendemicity. Note the break in the *y* axis between 10,000 and 50,000 RLU. (B.) Distribution of nonreactive S/CO values (S/CO of ≤1.0).

A total of 28,740 donor samples (19,532 plasma and 9,208 serum) were tested on the Architect using the prototype anti-Babesia assay. Upon analysis of the population, a provisional cutoff was set at 31.6 standard deviations from the median relative light unit (RLU) signal of the population (Fig. 1 and Table 1). There were seven initially reactive samples that were reactive when repeated (five plasma and two serum samples; initial/repeat reactive rate of 0.024%) when the provisional cutoff was applied to the population. The repeat reactive samples were further evaluated for B. microti antibodies by supplemental IFA testing (Table 2). Two repeat reactive plasma samples were confirmed by supplemental IFA testing; the resolved specificity was determined to be 99.98%. Analysis of the resulting S/CO distribution showed that 98.6% of samples had an S/CO of less than 0.1 (Fig. 1B). Additionally, a panel of antibody-positive cases of Lyme disease (*n* = 31) were tested, and none were found to be positive (see Table S1 in the supplemental material).

**TABLE 1:** Specificity of prototype anti-Babesia assay on donors from areas of nonendemicity

Parameter	Value by sample type^*a*^	
	Plasma (*n* = 19,532)	Serum (*n* = 9,208)
Sensitivity (RLU)		
Mean	156.18	137.30
Median	125.00	118.00
SD	165.52	126.90
95% CI	153.85–158.50	134.71–139.89
Cutoff^*b*^	5,000	
SD from median cutoff^*b*^	31.6	
Specificity (%)	99.97	99.99
Resolved specificity (% [95% CI])	99.98 (99.97–100.00)^*c*^	

a Total number of samples, 28,740.

b Determined for the combined population.

c Determined for both serum and plasma samples.

**TABLE 2:** Profile of repeatedly reactive samples

Sample type	Architect test (S/CO)			IFA test	
	Initial	Repeat reactive 1	Repeat reactive 2	Titer	Interpretation^*a*^
Plasma	35.6	36.2	36.2	1:128	Positive
	37.7	38.2	40.1	1:64	Positive
	36.6	39.4	37.9	<1:64	Negative
	12.7	12.9	13.5	<1:64	Negative
	1.6	1.5	1.5	<1:64	Negative
Serum	13.5	14.4	14.4	<1:64	Negative
	2.2	2.2	2.2	<1:64	Negative

a An IFA titer of ≥1:64 is considered positive.

### Dilutional sensitivity of the prototype anti-Babesia assay versus the IFA test.

The dilutional sensitivity of the prototype assay was compared to that of the IFA assay by evaluating the (CBER) Babesia antibody reference panel. Panel samples were tested as undiluted and at various 2-fold dilutions between 1:10 and 1:5,120 in the prototype assay or as undiluted in the IFA test (Table 3). Four of the 8 samples that had endpoint titers between 1:40 and 1:320 in the prototype assay were not detected by the IFA test. Among the four positive samples that were detected via IFA testing, comparison of final endpoint titers demonstrated that the prototype assay had better dilutional sensitivity than the IFA test and was 2.5- to 10-fold more sensitive (Table 3).

**TABLE 3:** Dilutional sensitivity of prototype anti-Babesia assay and IFA test

Sample no.	Final endpoint titer^*a*^	
	Architect assay	IFA test
1	1:80	<1:64
2	1:320	<1:64
3	1:5,120	1:512
4	1:640	1:128
5^*b*^	Negative	<1:64
6	1:1,280	1:512
7	1:160	<1:64
8	1:40	<1:64
9	1:2,560	1:512

a Each sample comprises a pool of IFA-reactive donors. For the anti-Babesia assay, the dilution shown is the last value to be considered positive in the assay (S/CO of >1.0). For samples detected by IFA, the titer indicated is the endpoint titer, and samples with titers of <1:64 are considered negative.

b Sample 5 was a negative control.

### Anti-Babesia assay sensitivity characterization.

**FIG 2:**
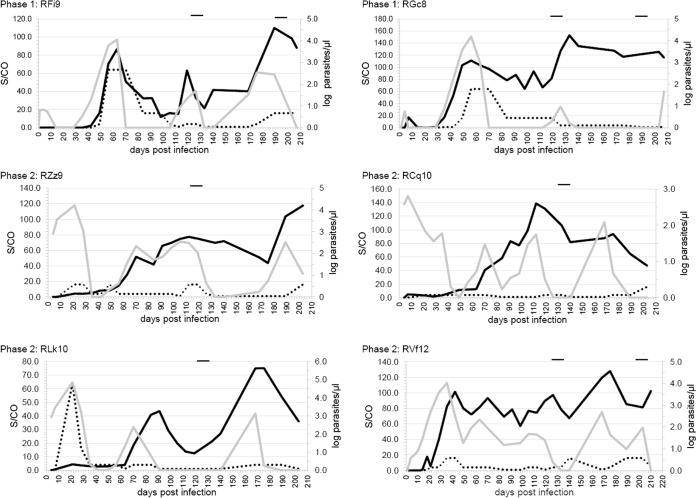
Comparison of anti-Babesia assay and IFA test S/CO values in macaque serial bleeds. Plots using the left *y* axes compare the S/CO values of the anti-Babesia assay (solid black line) and the IFA test (dotted line). The IFA test S/CO was determined by dividing the endpoint titer by the assay cutoff (1:64) for each time point. For example, a sample with an IFA titer of 1:64 had a corresponding S/CO of 1.0. Parasite abundance is indicated by the right *y* axes (gray line). Black bars at the top of the plots indicate drug treatment.

Serial bleed samples available from a macaque model of TTB were evaluated in the prototype anti-Babesia assay; results were compared to historic IFA test and PCR data (Table S2) (25). Historic data indicated that the first detection of parasite replication for the phase 1 macaques using hamster-passaged inoculum was approximately 35 days (peak detection, 56 to 63 days), and the first detection of parasite following inoculation with monkey-passaged B. microti (phases 2 and 3) occurred as early as 4 days (peak detections, 14 to 21 and 35 days, respectively) (Fig. 2 and Tables 4 and S2). The preseroconversion window period, defined as specimens testing negative via serology but detectable by PCR, lasted between 0 and 14 days (average, 7.5 days) prior to detection by the prototype anti-Babesia assay and between 0 and 18 days (average, 12.3 days) with the IFA test (Fig. 2 and Table S2). Detection of antibodies in phase 1 macaques using the prototype anti-Babesia assay occurred 7 days earlier (animal RGc8) or 7 days later (animal RFi9) than PCR detection (Fig. 2 and Tables 4 and S2). In phase 2 macaques, serologic detection using the prototype assay occurred at 17 days (animal RZz9), 3 days (RCq10), or 10 days (RLk10) after parasitemia was initially detected by PCR (Table 4). A total of 124/125 (99.2%) IFA-detected bleeds were positive in the prototype anti-Babesia assay, and three additional early seroconversion samples testing negative by IFA were detected using the prototype assay (Table 4).

**TABLE 4:** Performance of the prototype anti-Babesia assay for detection of antibodies in the macaque model of TTB

Phase and animal	Length of collection period (days)	Day of 1st PCR-positive result^*a*^	Day of 1st detection		Total no. of bleeds detected		No. of IFA-positive bleeds detected by the anti-Babesia assay (%)
			Anti-Babesia assay	IFA test	Anti-Babesia assay	IFA test	
Phase 1							
RFi9	207	35	42	49	19	18	18 (100)
RGc8	207	35	28^*b*^	35^*b*^	22	21	21 (100)
Phase 2							
RZz9	203	4	21	14	20	21	20 (95.2)
RCq10	203	4	7	14	22	21	21 (100)
RLk10	203	4	14	14	21	21	21 (100)
Phase 3							
RVf12	210	4	14	18	24	23	23 (100)
Total					128	125	124 (99.2)

a As reported in Gumber et al. (25).

b Macaque displayed transient immune response for a single bleed on day 7 (anti-Babesia assay) and on day 14 (IFA test). These results were not included in the total calculation.

Discordant results between the IFA test and the prototype anti-Babesia assay were observed during the early seroconversion period; the prototype assay detected antibodies earlier than the IFA test in three macaques (RFi9, 7 days; RCq10, 7 days; RVf12, 4 days) and later in one macaque (RZz9, 7 days) (Fig. 2 and Tables 4 and S2). Following seroconversion, both assays showed continued detection of antibodies throughout the duration of the study. The IFA assay had a strong initial response and then remained at (1:64; S/CO of 1.0) or near the cutoff (1:256) for the majority of the samples tested (Fig. 2), whereas the prototype assay had robust signal throughout.

Upon seroconversion, all subsequent samples from the macaques were detected by both antibody assays (Fig. 2 and Table S2). However, all phase 1 and phase 2 macaques demonstrated intermittent parasitemia, characterized as having recurring positive and negative PCR results, prior to and after the administration of atovaquone and azithromycin for 10 days (day 122) (Fig. 2 and Table S2). Overall, there were three macaques (animals RFi9, RGc8, and RLk10) that demonstrated intermittent parasitemia prior to drug administration. For the period prior to drug intervention, there were 17 samples that were negative for PCR but positive in both serologic tests (Tables 5 and S2). For the period after drug administration, all macaques became transiently negative by PCR for one or more bleeds, followed by a subsequent reemergence of parasitemia detected 6 weeks later by PCR in five of six macaques; antibody was detected in all bleeds (Table 5).

**TABLE 5:** Postseroconversion profile of parasitemia and antibody response in persistently infected macaques

Phase and animal	No. of positive antibody bleeds by treatment and PCR result			
	Prior to drug intervention		Post-drug intervention	
	PCR positive	PCR negative^*a*^	PCR positive	PCR negative^*a*^
Phase 1				
RFi9	6	3	5	2
RGc8	6	4	4	5
Phase 2				
RZz9	12	2	5	2
RCq10	14	1	2	2
RLk10	7	7	2	2
Phase 3				
RVf12	16	0	6	2
Total	61	17	24	21

a Samples that demonstrated PCR negativity and were seropositive in the anti-Babesia assay. Only samples that demonstrated return of PCR positivity were counted.

### Sensitivity of anti-Babesia assay of clinical babesiosis samples.

Samples previously characterized for babesiosis by both PCR and IFA testing (*n* = 58) were tested with the prototype anti-Babesia assay to determine sensitivity of clinical babesiosis samples. Positive concordance between the anti-Babesia assay by either PCR or IFA result was observed in 56/58 samples (96.55%; 95% confidence interval [CI], 87.6% to 99.7%) (see Table S3 in the supplemental material). The two discordant samples had IFA titers of 1:256 and 1:512, and no volume was available for repeat IFA testing. Subsequent testing for IgM in an indirect assay format demonstrated that 55/58 samples were IgM positive. One sample not detected in the prototype assay demonstrated low-level, but positive, IgM reactivity (Table S3).

## DISCUSSION

Transfusion-transmitted babesiosis (TTB) is the most frequently reported transfusion-associated infection in the United States. This study detailed the performance of a prototype antibody test for B. microti utilizing samples obtained from individuals considered at low risk for B. microti infection, from experimentally infected macaques, and from subjects diagnosed with active B. microti infection. The resolved specificity observed among blood donors was 99.98%. Further, the prototype assay detected 99.2% and 96.7% of previously characterized IFA-positive samples from experimentally infected macaques and human babesiosis cases, respectively. This study demonstrates a fully automated antibody assay employing recombinant antigens which can provide acceptable sensitivity and specificity to establish the seroprevalence of B. microti in various geographic regions and is a potential method to consider for screening blood donors for the presence of antibodies to *B. microti*.

One of the major challenges for a B. microti antibody test is to provide acceptable specificity when testing in regions where exposure to the parasite is considered nonendemic. Donors with reactive results would be unnecessarily deferred due to a false-positive result. A direct antibody assay format was selected to reduce the number of false-positive results compared to the number of results with an indirect format and to detect both IgM and IgG class antibodies (29, 30). Using this format, we observed large signal separations between known negative and positive samples, as shown in Fig. 1, where 98.6% of the samples considered to be at low risk for B. microti infection had S/CO values of <0.10. Using the provisional cutoff set at approximately 32 standard deviations from the population mean, only 7 of 28,740 samples sourced from areas of nonendemicity were repeatedly reactive in the prototype test. Supplemental IFA testing detected two of the seven repeatedly reactive samples; thus, the resolved specificity was determined to be 99.98%. While antibody testing alone limits the ability to distinguish between recent or past infection, whole blood was not available from repeatedly reactive donors for subsequent PCR testing. The specificity value was comparable to values of investigational assays seeking licensure for blood screening in the United States (8, 21, 22) but superior to previous reports using IFA testing, where specificity values ranged from 90.0 to 100.0% (11). In summary, the resolved specificity values were compatible with specificity expectations for a prospective B. microti antibody test (14).

The prototype assay employed two distinct B. microti-specific antigens, BMN1-9 and BMN1-17, which were previously shown to be immunodominant (26–28). A comparison of IFA testing and immunoassay using recombinant proteins found sensitivity estimated to be between 98.0% (26) and 98.7% (28), and an enzyme-linked immunosorbent assay (ELISA) using peptides derived from BMN1-17 and BMN1-9 had a sensitivity of 91.1% (21, 31). In the present study, performance data showed an assay comprised only of BMN1-9 and BMN1-17 had a detection rate comparable to that of IFA testing, which utilizes the whole parasite, suggesting that the immunodominant epitopes represented in the recombinant proteins are sufficient to detect the vast majority of seropositive samples. For experimentally infected macaques, 124 of 125 (99.2%) samples detected as IFA positive were positive in the prototype antibody assay. Among four discordant samples noted, all were observed during the early seroconversion period, with three detected only by the prototype assay and one detected only by IFA testing; the results indicate that the prototype assay provides equivalent or better sensitivity than IFA testing. We noted that the prototype anti-Babesia assay and IFA assays appeared to have similarly robust responses early in infection, but in later stages of infection (3 to 6 months postinfection), the prototype assay provided robust signals compared to results of the IFA test. In comparing dilutional sensitivities on a panel of samples from seropositive human subjects available through the FDA, the prototype assay detected samples diluted between 1:40 and 1:1,280 and was determined to be 2.5- to 10-fold more sensitive than the IFA test. For clinical babesiosis samples characterized as being positive both by PCR and IFA test, the prototype assay detected 56 of 58 samples. Additionally, it is possible that assay interference may have played a role in discordant results between the anti-Babesia assay and the IFA test; although not evaluated here, assay interference will be pursued as part of the assay development process. The clinical babesiosis samples were preselected as IFA positive, so it is possible that with expanded prospective studies, there may be parasitemic human subjects who are detected with the prototype assay but negative by IFA test.

As noted with other blood-borne pathogens such as HIV, hepatitis B virus (HBV), and hepatitis C virus (HCV), during early infection there is a preseroconversion window period requiring the detection of pathogen-specific nucleic acid or proteins to diagnose infection (22). For phase I macaques, low-level parasitemia was observed in the 1 to 7 days following infection, which was likely due to the inoculum (10^9^ parasites) as this phase was followed by >2 weeks of PCR negativity. In the macaques, the duration of the preseroconversion period (PCR positive and antibody negative) was observed to be between 0 and 14 days (average, 7.5 days) using the prototype assay and between 0 and 18 days (average, 12.3 days) using the IFA test. During this period, there were a total of 10 PCR-positive samples not detected by the prototype and 13 not detected with IFA test. Recently, 15 preseroconversion window-period samples testing positive with molecular tests for Babesia were identified in a study screening volunteer blood donors in areas of endemicity using both molecular and arrayed fluorescence assays (1 per 15,000 donations) (32). In these cases, the duration of the window period could not be determined, but most donors participating in follow-up (average, 1.6 months later) seroconverted. These observations support the need for combined testing with a reliance on nucleic acid testing to detect window-period samples and antibody testing to detect nonacute donors with low-level (undetectable) parasitemia (8).

Following the window period, seroconversion occurred in all six macaques when they were tested by both IFA and the prototype assay, and antibody detection continued throughout the study period. In the early days after seroconversion and prior to the administration of drugs, 17 samples from five seropositive macaques had undetectable parasitemia by PCR. In all cases, the period of undetectable parasitemia was followed by positive PCR results, indicating that the macaques were persistently infected even though infection was not detected by PCR (Fig. 2 and Table S2 in the supplemental material). Although infection was not detectable by the PCR testing described here, these macaques may be considered carriers and could possibly transmit infection. It has been reported that seropositive donors testing as PCR negative may be able to transmit infection to hamsters (8). Persistent parasitemia for more than 6 months has been previously observed in naturally occurring infections of nonhuman hosts (33), in experimentally infected animals (25), and in human subjects (8, 34–37). There are limited reports of recrudescence in human infections, most occurring in immunocompromised individuals (36). It is possible that recrudescence is underestimated because follow-up testing is not pursued once a person tests negative via PCR. As more-sensitive molecular tests are developed, there will be fewer instances of active B. microti infection in seropositive subjects testing negative by PCR. Minimally, serologic testing can detect persistently infected B. microti carriers that show periodic negative results by PCR.

Although 99.2% of macaque IFA-positive samples were detected with the prototype anti-Babesia assay, the dynamics of antibody response were different between the two assays. For the IFA test, within a few weeks after seroconversion, the antibody signals for parasitemic samples from macaques remained borderline reactive in contrast to the robust signals of the prototype anti-Babesia assay. Similarly, in human infections, transient seropositivity occurs as assessed by the IFA test, where loss of IFA titer (<1:64) is followed by a return to a low-level positive result in subsequent bleeds (37). In one case, both PCR and IFA testing failed to detect infection in selected bleeds of a persistently infected patient (37). Few studies of human Babesia infection have longitudinal samples evaluated using both PCR and IFA testing. In two studies (8, 37), subjects were followed using PCR and IFA testing for several months following index collection, and selected subjects displayed transient seropositivity using the IFA test. Although we do not know the performance of the prototype anti-Babesia assay on these samples, these two studies support discrepancies in IFA detection of true seropositive samples.

The current estimated risk of TTB among blood donors in areas of endemicity is 1 per 101,000, which is much greater than the risks for HIV and HCV (1 per 1,020,000 and 1 per 218,000, respectively) (8). Unless measures are taken to prevent TTB, the number of cases is likely to increase as the number of reported cases of babesiosis is increasing. Data presented here and in at least one other study (27) suggest that neither NAT nor serology testing alone is sufficient to mitigate TTB in the United States, and optimal screening strategies that take advantage of more sensitive diagnostic tools are needed.

## Supplementary Material

Supplemental material
