# Supplementary material for: Performance Evaluation of a Prototype Architect Antibody Assay for Babesia microti
Source: J Clin Microbiol. 2018 Jul 26;56(8):e00460-18. doi: 10.1128/JCM.00460-18 (PMC6062809; doi:10.1128/JCM.00460-18)
Supplement: Supplemental material [file supp_56_8_e00460-18__index.html]

Supplemental material 

# Performance Evaluation of a Prototype Architect Antibody Assay for Babesia microti

## Supplemental material

- Supplemental file 1 -

  Table S1 (Evaluation of Lyme-diagnosed samples in anti-*Babesia* assay)

  XLSX, 9.1K
- Supplemental file 2 -

  Table S2 (*Babesia microti*-infected macaque seroconversion samples [S/CO of ≥1.0 is considered positive])

  XLSX, 18K
- Supplemental file 3 -

  Table S3 (Clinical babesiosis sample testing [S/CO of ≥1.0 is considered positive])

  XLSX, 12K
